# Verrucous carcinoma of the esophagus: a case report and literature review

**DOI:** 10.1186/s40792-020-0801-8

**Published:** 2020-02-07

**Authors:** Satoshi Tabuchi, Kazuo Koyanagi, Koji Nagata, Soji Ozawa, Shigeyuki Kawachi

**Affiliations:** 1 Department of Digestive Surgery and Transplantation Surgery, Tokyo Medical University Hachioji Medical Center, 1163 Tatemachi, Hachioji, Tokyo, 193-0998 Japan; 2 Department of Gastroenterological Surgery, Tokai University School of Medicine, 143 Shimokasuya, Isehara, Kanagawa 259-1193 Japan; 3 Department of Pathology, Saitama Medical University International Medical Center, 1397-1 Yamane, Hidaka, Saitama, 350-1298 Japan

**Keywords:** Esophageal carcinoma, Verrucous carcinoma, Thoracoscopic surgery

## Abstract

**Background:**

Verrucous carcinoma is an extremely rare form of cancer in the esophagus.

**Case presentation:**

A 56-year-old woman presented with dysphagia in 2007. Endoscopic examination revealed an irregular protruding circumferential erosion in the lower thoracic esophagus, but because pathological examination of the biopsy specimen showed no evidence of malignancy, the status of the erosion was followed up by an upper gastrointestinal endoscopic examination every 3 months. A year later, polypoid lesions and fungal infection were observed in the eroded area, but no evidence of malignancy was detected in the biopsy specimen at the time. Eighteen months later, the polypoid lesions had increased in size, and the biopsy specimen was diagnosed as highly suspicious of well-differentiated squamous cell carcinoma. Because the patient’s condition deteriorated due to worsening of the dysphagia and weight loss, we performed a thoracoscopic esophagectomy with lymph node dissection and reconstructed the alimentary tract with a gastric tube via the posterior mediastinal route. Macroscopic examination of the resected specimen showed a white protruding lesion with an irregular surface, and histopathological examination led to a diagnosis of esophageal verrucous carcinoma without lymph node metastasis. No signs of recurrence have been observed in the 8 years since surgery.

**Conclusion:**

We have reported a long-term follow-up case of verrucous carcinoma of the esophagus that was difficult to diagnose before surgery.

## Background

Verrucous carcinoma (VC) was first described as a variant of squamous cell carcinoma by Ackerman in 1948 [1] and is characterized as a slow growing, well-differentiated, locally spreading tumor. However, it is very difficult to diagnose as VC before surgical excision, because in most cases, the superficial layer of the tumor is covered by non-malignant tissue [2]. We report a surgical case of VC of the esophagus in which a long period was required to make the diagnosis, and we include a review of the literature.

## Case presentation

A 56-year-old woman presented with dysphagia in 2007. The results of an endoscopic examination performed at the previous hospital were unremarkable. Her complaints became more severe in November 2009, and she was referred to our hospital for treatment.

### Initial medical examination

**Fig. 1:**
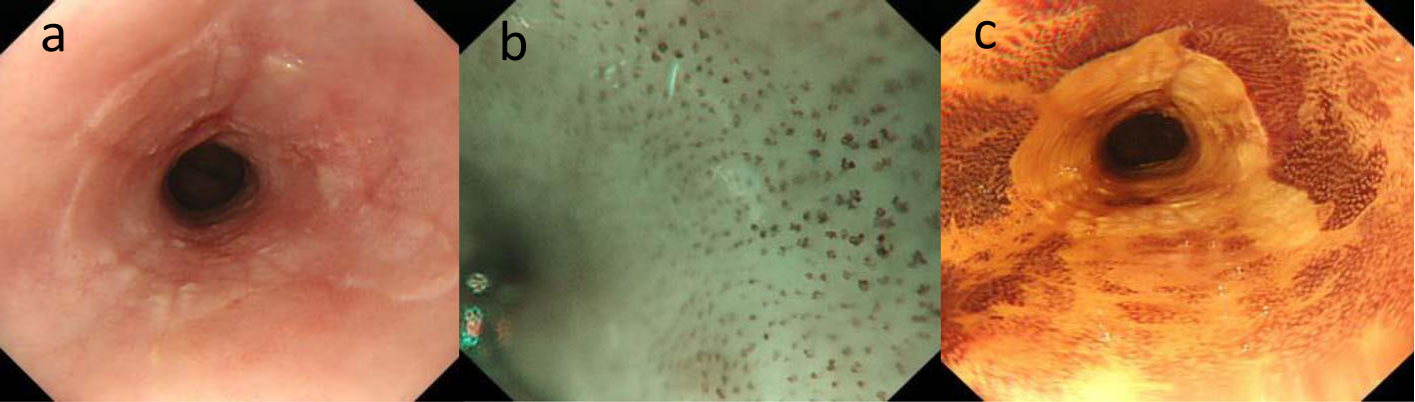
**a** Endoscopic examination showed circumference erosions on the esophageal mucosa at 28-40 cm from the incisors. **b** Type A vessels were detected by magnified narrow band imaging (NBI). **c** The lesion was not stained by iodine, but malignant findings were not detected at biopsy specimen

**Fig. 2:**
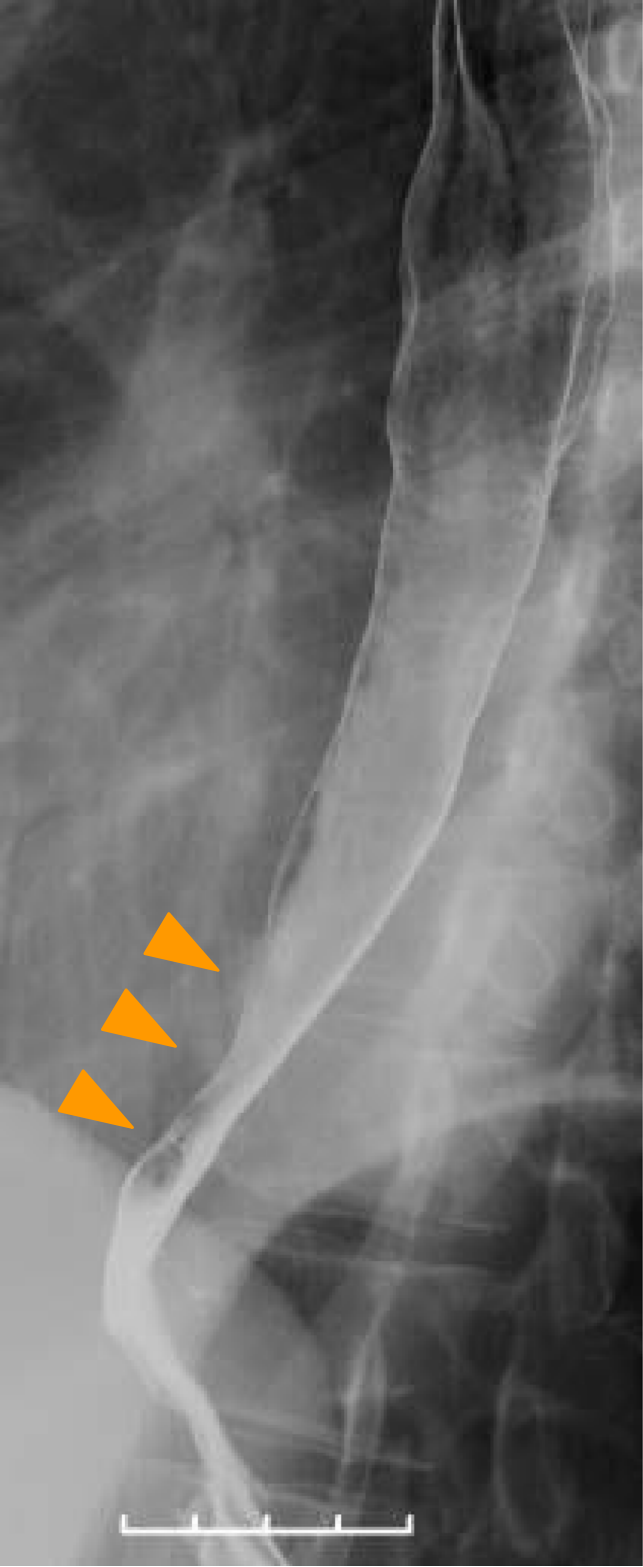
Esophagography revealed the smooth stenosis at the lower esophagus (arrow heads)

Endoscopic examination at our institution revealed a circumferential erosion of the esophageal mucosa 28-40 cm from the incisors, and it was impossible to advance the endoscope due to stenosis at 37-40 cm (Fig. 1a). Type A vessels were detected by magnified narrow band imaging (NBI) (Fig. 1b), and the lesion did not stain with iodine (Fig. 1c). Esophagography revealed smooth stenosis of the lower esophagus (Fig. 2). A CT scan showed mild thickening of the lower thoracic esophageal wall but no evidence of lymph node swelling in the thoracic or abdominal cavity. 18F-fluorodeoxyglucose (FDG) positron emission tomography (PET)/computed tomography (CT) did not show FDG uptake, and the levels of the tumor markers SCC, CEA, and anti-p53 antibody were within their normal ranges. Although the lesion was suspected of being a malignant tumor of the esophagus, pathological examination of biopsy specimens from four different sites resulted in a diagnosis of esophagitis. We then performed esophageal high-resolution manometry and an esophageal pH study, but because these studies did not reveal abnormal esophagogastric reflux or esophageal motor dysfunction, we followed up the lesion by upper gastrointestinal endoscopy every 3 months.

### Follow-up examination at 1 year

**Fig. 3:**
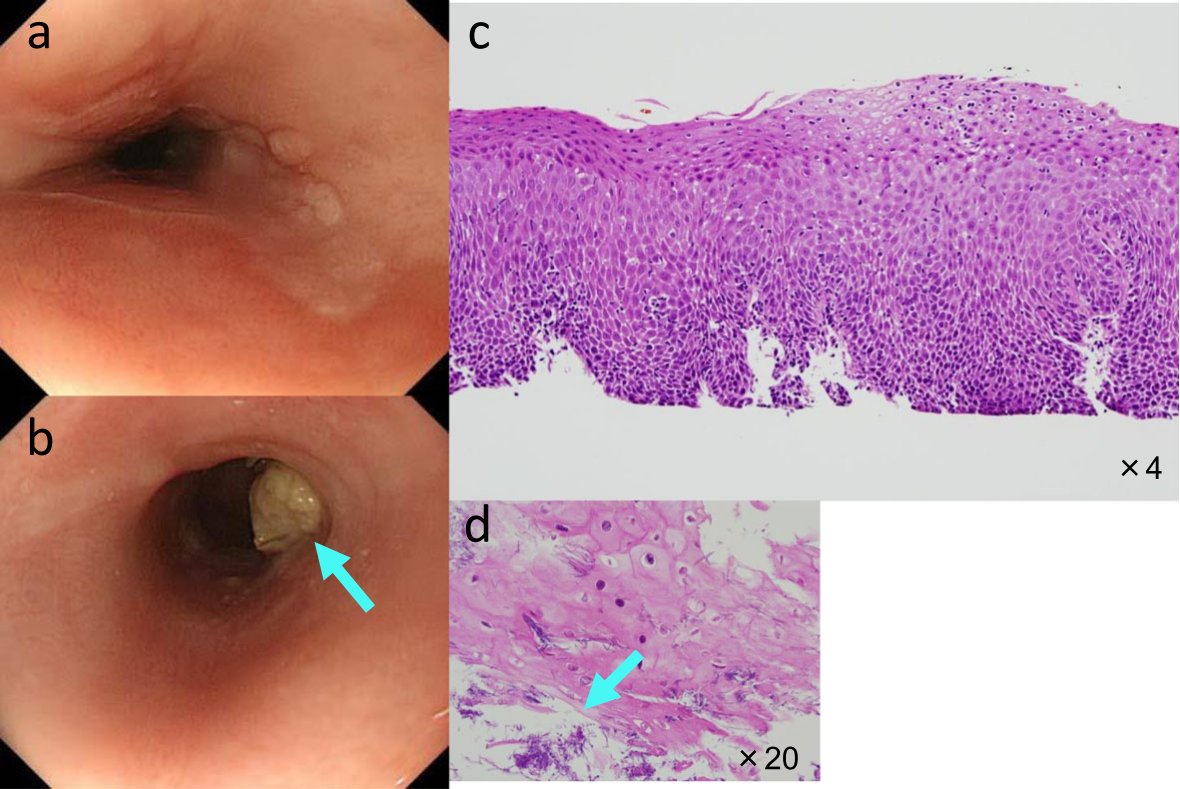
Endoscopic examination 1 year later. **a**, **b** Polypoid lesions with fungal infection appeared on the erosive lesion (arrows). **c** Malignant findings were not recognized at biopsy specimens. **d** Fungal infection was observed on the epithelial surface of the biopsy specimens (arrows)

Polypoid lesions and fungal infection were observed on the erosive lesion during the follow-up examination after 1 year (Fig. 3a, b), but no evidence of malignancy was detected during the pathological examination of the biopsy specimens (Fig. 3c, d), and the CT scan showed no particular changes during the period. Antifungal agents were administered for 1 month for fungal infection of the lesions, but the lesions did not change.

### Follow-up examination at 18 months after the initial examination

**Fig. 4:**
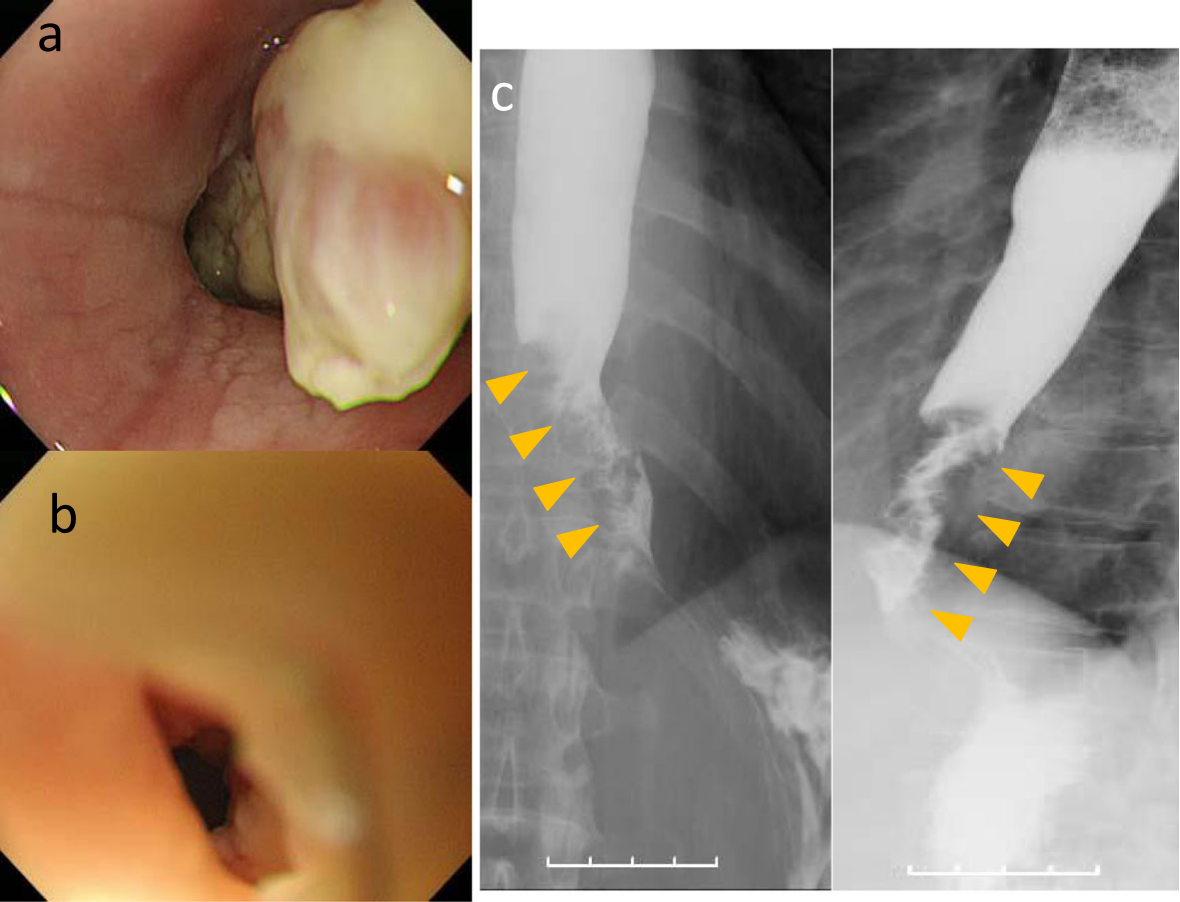
Endoscopic examination and esophagography 1 year and 6 months later. **a**, **b** Polypoid lesions increased further, and the biopsy specimen was diagnosed as neoplasia. **c** Esophagography revealed the irregular stenosis at the lower esophagus (arrow heads)

**Fig. 5:**
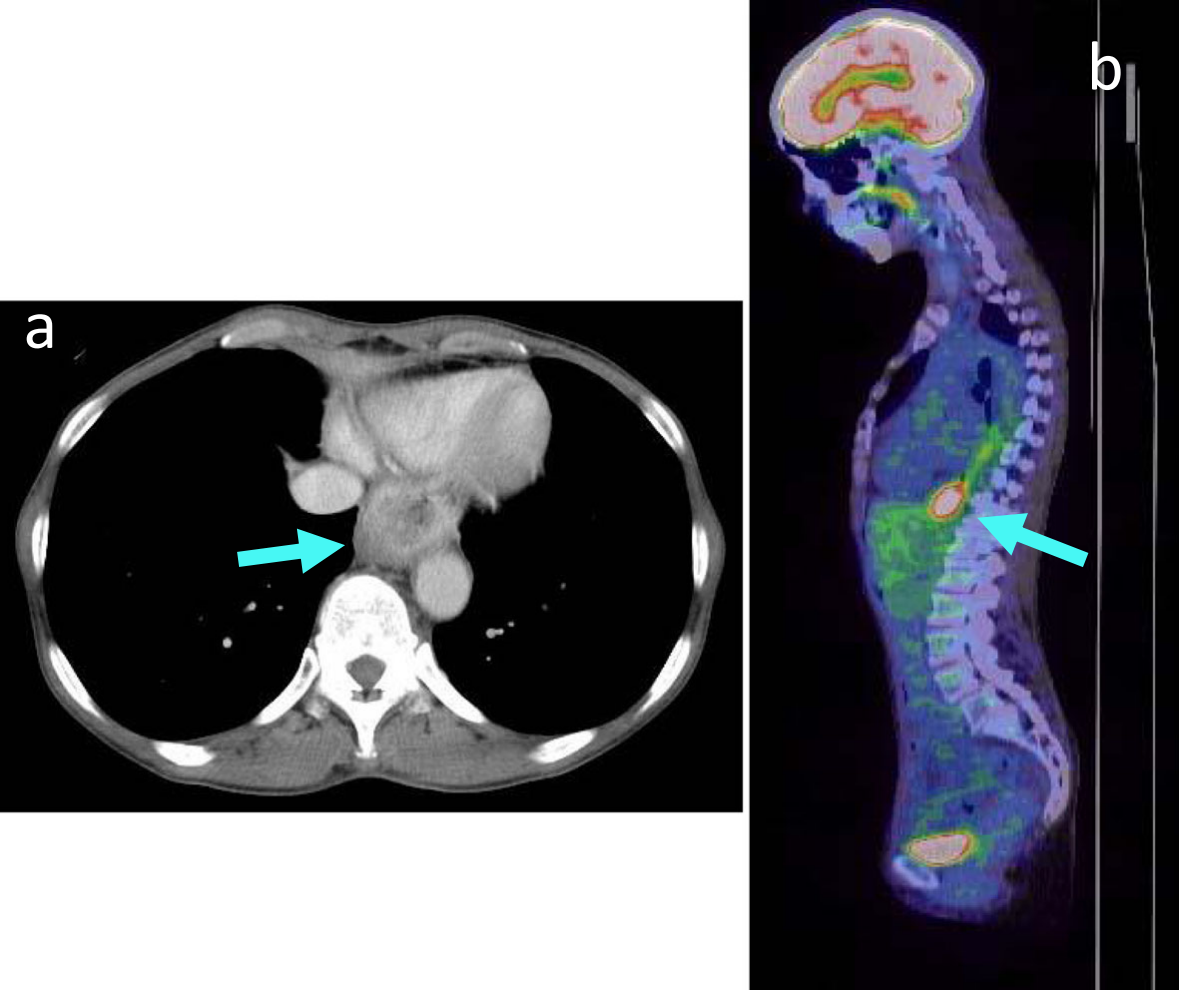
CT and PET/CT 1 year and 6 months later. **a** CT scan found the obvious wall thickening at the lower esophagus (arrows). **b** PET/CT showed increased FDG uptake in the lower esophagus (SUV max, 9.5) (arrows)

Polypoid lesions have further increased in size (Fig. 4a, b), and the biopsy specimen was diagnosed as highly suspicious of well-differentiated squamous cell carcinoma. Esophagography revealed irregular stenosis (Fig. 4c), and a CT scan showed circumferential wall thickening in the lower esophagus (Fig. 5a) with increased FDG uptake (SUV max, 9.5) on PET/CT (Fig. 5b). Because the patient’s condition rapidly deteriorated due to worsening of the dysphagia and weight loss, we performed a thoracoscopic esophagectomy with lymph node dissection and reconstruction with a gastric tube via the posterior mediastinal route. The postoperative course was uneventful, and the patient was discharged on postoperative day 28. The patient did not receive any adjuvant chemotherapy, and there have been no recurrences as of 8 years after surgery.

### Pathological findings

**Fig. 6:**
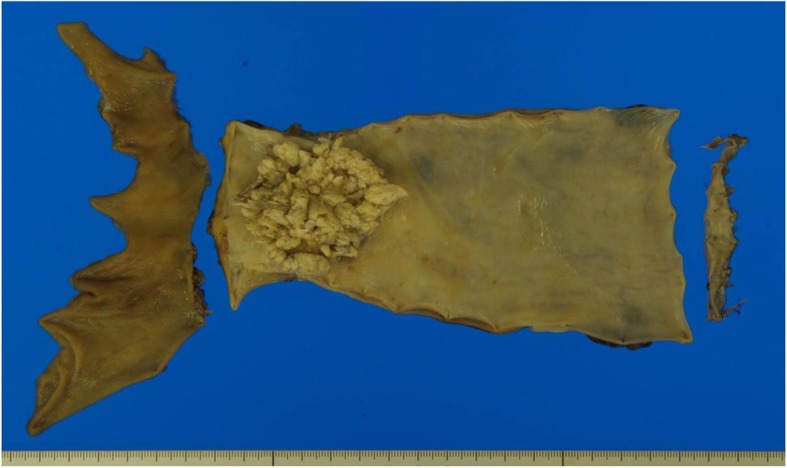
Macroscopic findings of the resected specimen. White protruding lesion with an irregular surface was seen at the lower esophagus

**Fig. 7:**
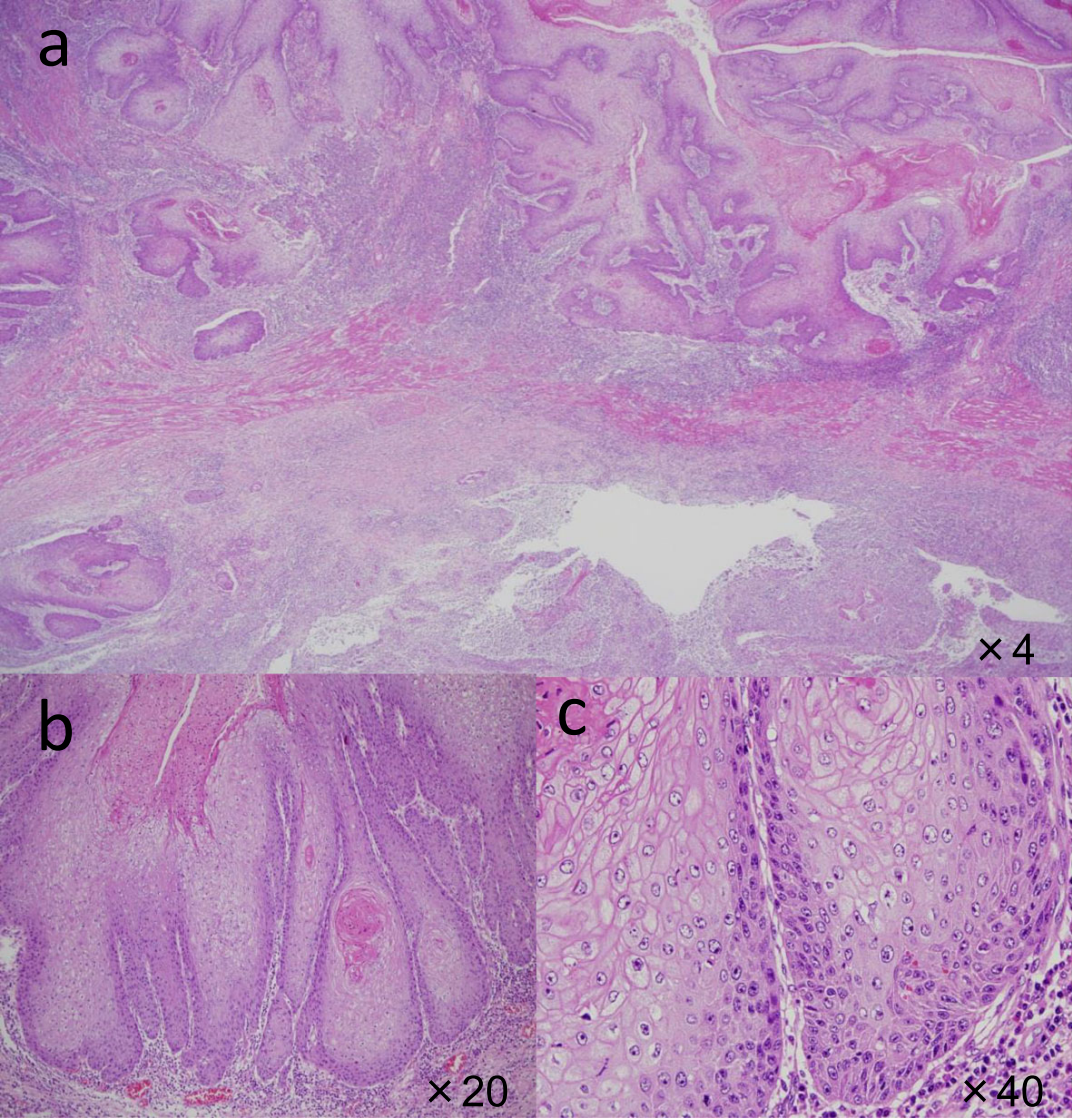
Microscopic findings of the tumor. **a** Microscopic findings showed epithelial downgrowth and invasive findings and diagnosed as well-differentiated squamous cell carcinoma. **b**, **c** Focal hyperkeratosis with a church spire configuration was seen

Macroscopic examination of the resected specimen revealed a white protruding lesion with an irregular surface in the lower esophagus (Fig. 6). Microscopic examination showed epithelial downgrowth and invasive findings of the tumor, and the diagnosis was xwell-differentiated squamous cell carcinoma. Focal hyperkeratosis with a church spire configuration was also seen (Fig. 7). These macroscopic and microscopic features were consistent with the growth pattern of VC. The pathological stage was T3N0M0, pStageIIA (UICC7th). No venous or lymphatic invasion was detected. All surgical margins were negative for malignancy.

## Discussion

Verrucous carcinoma (VC) is an exophytic, warty, and cauliflower-like tumor and a slowly growing, well-differentiated variant of squamous cell carcinoma. VC rarely metastasizes to lymph nodes and distant organs. It is found most commonly in the oropharynx, genitalia, and the soles of the feet [3–5]. VC of the esophagus is very rare [6] and difficult in preoperative diagnosis. A PubMed search using the keywords “esophagus” and “verrucous carcinoma” retrieved 38 cases during the period from 1983 to 2018, and the details are shown in Table 1 [7–14]. The patients ranged in age from 36 to 78 years old (median = 63). Males predominated (23 cases versus 13, with the gender in 2 unknown). The most common chief complaint was dysphagia, in 30 out of the 38 cases (79%). The tumor was located in the upper esophagus in 9 cases, the mid-esophagus in 7 cases, and in the lower esophagus in 20 cases. In two cases, the tumor involved the entire esophagus.

**Table 1:** Clinical characteristics of verrucous carcinoma of the esophagus

Age	63 (36~78)
Sex	M, 23
	F, 13
	Unknown, 2
Chief complaint	Dysphagia, 30
	Chest pain, 3
	Melena, 3
	No symptom, 1
	Unknown, 1
Tumor location	Ut, 9
	Mt, 7
	Lt, 20
	Whole, 2
Past history	Achalasia, 5
	Reflex esophagitis, 9
	Diverticulum, 2
	Obstruction, 2 (drug, 1; traumatic, 1)
	Candida esophagitis, 1
	None, 19
Initial diagnosis	Benign, 20
	VC, 10
	SCC, 5
	Unknown, 3
Tumor size	≥ 5 cm, 25
	< 5 cm, 5
	Unknown, 8
Treatment	Operation, 26
	Irradiation, 2
	Chemotherapy, 2
	Conservative, 8
pT	T1, 9
	T2, 9
	T3, 4
	T4, 8
	Unknown, 8
pN	Negative, 24
	Positive, 3
	Unknown, 11
pM	Negative, 25
	Positive, 0
	Unknown, 13
Prognosis	Dead, 15
	Alive, 15
	Unknown, 8

There was a previous medical history of esophageal achalasia in 5 cases, reflux esophagitis in 9 cases, esophageal diverticulum in 2 cases, esophageal stricture in 2 cases, and candida esophagitis in 1 case. Based on these medical histories, retention of esophageal content might result in chronic esophageal inflammation and induce the development of esophageal VC [11]. In our own patient, candida esophagitis was diagnosed during the follow-up period and might be related to the esophageal VC. There are also a number of reports of human papilloma virus (HPV) being related to primary laryngeal VC. Liberale et al. have reported HPV-positive esophageal VC cases [14]. In 25 of the 30 cases in which tumor size was recorded, the tumor measured 5 cm or more in diameter. Tumor size was relatively large, but depth of invasion was generally shallow. Lymph node metastasis was present in 3 out of the 27 cases in which it was mentioned. Moreover, the shape of the tumors was unique, with the papillary elevations often compared with cauliflowers.

Although histopathological diagnosis of esophageal VC is difficult, the histopathologic features of esophageal VC include good preservation of the epithelial basement membrane and highly differentiated histology, which are important to differentiating esophageal VC from other esophageal carcinomas. It is also difficult to differentiate esophageal VC from esophageal papilloma: Esophageal VC tends to be deep-growing and invasive, whereas papilloma tends to grow superficially. Oh et al. has reported that endoscopic mucosal resection (EMR) might be essential to accurate diagnosis in cases of suspected esophageal VC [2]. Therefore, for the earlier and accurate diagnosis of this type of tumor, it is important to obtain a large piece of tissue by EMR or endoscopic submucosal dissection. However, because of the limited low-grade nevus cell nest formation and the highly keratinized surface of the tumors, it is not easy to diagnose esophageal VC in preoperative biopsy samples. In fact, only 10 of 38 cases (26%) were diagnosed as esophageal VC based on the biopsy pathology findings. In our own patient, no diagnosis of malignancy on the circumferential erosion was made at the first examination. The lesions had worsened greatly in approximately 18 months, and neoplasia was confirmed in an endoscopic biopsy specimen. When malignancy is suspected, regular endoscopic checkups and observation should be considered, as in our own patient.

Treatment consisted of surgery in 26 cases, radiation therapy in 2 cases, chemotherapy in 2 cases, and conservative treatment in 8 cases. Surgical treatment after 1990 provided desirable results because of improvements in surgery in recent years as well as because of the low malignant features of esophageal VC. On the other hand, opinions about the effectiveness of radiation therapy vary. Both Gothals [4] et al. and Kraus [5] et al. have respectively shown that radiation is not effective and that recurrence and early metastasis with anaplastic transformation tend to occur after radiation therapy. Previous reports have stated that chemotherapy might be inadequate as a means of curative therapy.

## Conclusion

We have reported a long-term follow-up case of VC of the esophagus that was difficult to diagnose preoperatively. Since distant or lymph node metastasis with localized growth are relatively rare in esophageal VC, we strongly believe that surgery, which can enable long-term survival possible, should be considered first, with curative intent.

## Data Availability

Data sharing is not applicable to this article, as no datasets were generated or analyzed during the current study.

## Abbreviations

EMR: Endoscopic mucosal resection
HPV: Human papilloma virus
NBI: Narrow band imaging
VC: Verrucous carcinoma
